# Multidisciplinary treatment for patients with chronic kidney disease in pre-dialysis minimizes costs: a four-year retrospective cohort analysis

**DOI:** 10.1590/2175-8239-JBN-2020-0226

**Published:** 2021-04-12

**Authors:** Celso Souza de Moraes, Natália Maria da Silva Fernandes, Fernando Antônio Basile Colugnati

**Affiliations:** 1 Universidade Federal de Juiz de Fora, Programa de Pós-Graduação em Saúde Brasileira, Juiz de Fora, MG, Brasil.

**Keywords:** Renal Insufficiency, Chronic, Predialysis, Dialysis, Costs and Cost Analysis, Health System, Insuficiência Renal Crônica, Prédiálise, Diálise, Custos e Análise de Custo, Sistema de Saúde

## Abstract

**Introduction:**

Chronic kidney disease (CKD) can progress to end-stage renal disease (ESRD), and clinical studies show that this progression can be slowed. The objective of this study was to estimate the costs to Brazil’s public health system (SUS) throughout the course of CKD in the pre-dialysis stage compared to the costs to the SUS of dialysis treatment (DT).

**Methods:**

A retrospective cohort study was conducted to analyze clinical and laboratory variables; the outcome analyzed was need for DT. To assess cost, a microcosting survey was conducted according to the Methodological Guidelines for Economic Evaluations in Healthcare and the National Program for Cost Management, both recommended by the Brazilian Ministry of Health for economic studies.

**Results:**

A total of 5,689 patients were followed between 2011 and 2014, and 537 met the inclusion criteria. Average costs increased substantially as the disease progressed. The average cost incurred in stage G1 in Brazilian reals was R$ 7,110.78, (US$1,832.06) and in stage G5, it was R$ 26,814.08 (US$6,908.53), accumulated over the four years.

**Conclusion:**

A pre-dialysis care program may reduce by R$ 33,023.12 ± 1,676.80 (US$ 8,508.26 ± 432.02) the average cost for each year of DT avoided, which is sufficient to cover the program’s operation, minimizing cost. These results signal to public health policy makers the real possibility of achieving significant cost reduction in the medium term for CKD care (4 years), to a program that disbursed R$ 24 billion (US$ 6.8 billion) for DT in Brazil between 2009 and 2018.

## Resumo

**Introdução:**

Doença renal crônica (DRC) pode progredir para doença renal estágio terminal (DRET). Estudos clínicos mostram que esta progressão pode ser retardada.

**Objetivo:**

estimar custos para o sistema público de saúde (SUS) do Brasil durante o curso da DRC no estágio pré-diálise, comparado com os custos para o SUS do tratamento dialítico (TD).

**Métodos:**

Conduziu-se estudo de coorte retrospectivo para analisar variáveis clínicas e laboratoriais; o desfecho analisado foi a necessidade de TD. Para avaliar os custos, realizou-se pesquisa de microcustos de acordo com as Diretrizes Metodológicas para Avaliações Econômicas em Saúde e o Programa Nacional de Gestão de Custos, ambos recomendados pelo Ministério da Saúde Brasileiro para estudos econômicos.

**Resultados:**

Acompanhou-se um total de 5.689 pacientes entre 2011-2014; 537 preencheram os critérios de inclusão. Os custos médios aumentaram substancialmente à medida que a doença progrediu. O custo médio incorrido no estágio G1 em reais foi R$ 7.110,78 (US$ 1.832,06) e no estágio G5 foi R$ 26.814,08 (US$ 6.908,53), acumulado durante os quatro anos.

**Conclusão:**

Um programa de atendimento pré-dialítico pode reduzir em R$ 33.023,12 ± 1.676,80 (US$ 8.508,26 ± 432,02) o custo médio para cada ano de TD evitado. Isso é suficiente para cobrir a operação do programa, minimizando custos. Estes resultados sinalizam aos formuladores de políticas de saúde pública a possibilidade real de alcançar redução significativa de custos em médio prazo para o cuidado da DRC (4 anos), para um programa que desembolsou R$ 24 bilhões (US$ 6,8 bilhões) para TD no Brasil entre 2009-2018.

## Introduction

The International Society of Nephrology estimated in a recent publication that approximately 10% of the world population lives with chronic kidney disease (CKD). CKD can progress in various ways to end-stage renal disease (ESRD), and clinical studies show that the progression of CKD to ESRD can be slowed. Despite well-established preventive strategies, thousands of people live with ESRD^1^. Approximately 0.1% of the world population has ESRD, and estimates suggest that the prevalence is higher in medium-high (0.1%) and high (0.2%) income countries compared to low (0.05%) or medium-low (0.07%) income countries^2^.

According to the Brazilian dialysis census, which publishes annually the number of patients undergoing dialysis in the country, in 2018 there were 133,464 patients undergoing dialysis. Eighty percent of these patients are funded by the Unified Health System (Sistema Único de Saúde - SUS)^3^, Brazil’s public health system, as determined by the Brazilian constitution of 1988 and implemented in 1990, which states that “health is a right of all and duty of the state”^3^. Data from the Brazilian Society of Nephrology and other researchers^4^^,^ 5 confirm the historic increase in the demand for dialysis treatment (DT) services.

The increase in CKD in Brazil does not yet seem to be a reason for more aggressive health policy actions. Data on the prevalence of the disease worldwide and from other studies point to a marked increase in CKD, including in children^3^^,^ 6 ^-^ 9. According to the study by Vanholder et al.^14^, the care of patients with CKD during the progression of the disease, i.e., treating the main causes (in the context of primary prevention) or progression and complications (secondary prevention), is still an underexplored field, despite the great potential to significantly reduce the social cost of CKD. Unfortunately, studies have indicated that in recent years, health policies have been more focused on treatment than prevention^15^^,^ 16. In this sense, treatment strategies during predialysis stages that delay the need for DT, acting in the preventive and periodic monitoring of patients who have some moderate to high epidemiological risk factor^15^^-^ 18, are effective.

Therefore, it is pertinent to the Brazilian context to understand the cost of reimbursing predialysis specialized care service providers, considering the possible avoidable costs with DT service providers. Thus, this study focuses on the cost of care in monitoring the stages of CKD progression in a predialysis outpatient clinic setting compared to the costs of DT to the public health system. The study intends to determine the cost savings with DT service providers from the establishment of predialysis monitoring actions in the medium term.

The objective of this study was to estimate the SUS costs from service providers over the course of CKD in predialysis care and compare with the costs of DT service providers.

The present study is relevant to the context of public policies for combating CKD and its economic impact amid fiscal adjustment policies, considering the possibility of delaying the entry of patients with CKD into the DT phase, thus increasing the possibility of saving public resources^19^^,^ 20.

## Materials and Methods

### Data sources

This longitudinal retrospective observational study involved the collection of data from medical records of patients seen at a clinical center specializing in predialysis care associated with the public health program of the state of Minas Gerais, Brazil, serving 37 cities. The center focused on secondary preventive care for diabetes, hypertension, and CKD, considering as medical specialists: nephrologists, cardiologists, and endocrinologists. In addition, there is a multidisciplinary team that assist the patient in a “circular” model (in the same outpatient consultation), which includes nurses, nutritionists, psychologists, social workers, pharmacists, dentists, physical educators, and physiotherapists. Data collection was authorized by the Ethics Committee of the Federal University of Juiz de Fora (Universidade Federal de Juiz de Fora - UFJF) and approved under protocol no. 36345514.1.0000.5139.

### Inclusion criteria and study period

The initial sample included 5,689 patients followed-up between 2011 and 2014 who were seen at all outpatient clinics. Inclusion criteria were patients seen at the nephrology outpatient clinic, independent of visits at the endocrinology and/or cardiology outpatient clinics. Exclusion criteria included patients treated before 2010 and after 2014 and patients in CKD stages G1 to G4 who stopped participating in the program between 2011 and 2014. Patients in stage G5 who stopped participating in the program were noted as patients who started DT. It was not possible to determine if these patients were dead or alive.

Data were obtained for 537 patients. Sociodemographic data, CKD progression stage, comorbidities (hypertension and diabetes), number of specialized medical consultations, and probable outcomes of referral to DT were collected from the medical records. Regarding the data on CKD progression, the probabilities of transition between disease stages were calculated according to the Kidney Disease: Improving Global Outcomes (KDIGO) criteria^21^. 

### Outcome measures

The center was funded by the state of Minas Gerais, which made fixed fund transfers to cover the monthly cost of the care provided by the service provider. Accordingly, the values of the transfers from the Minas Gerais State Health Fund (Fundo Estadual de Saúde de Minas Gerais - FES-MG) to the center were determined, and the average cost per patient was calculated, estimated by the total number of specialized medical consultations performed.

To validate the cost of the service provider, a microcosting survey was conducted following the Methodological Guidelines for Economic Evaluation in Health (Diretrizes Metodológicas para Avaliação Econômica em Saúde) and the National Program for Cost Management (Programa Nacional de Gestão de Custos - PNGC), both recommendations published by the Brazilian Ministry of Health^22^^-^ 24 for economic studies. 

The microcosting calculation was performed based on data from the retrospective financial database of the outpatient center to determine whether there was any restriction of costs by the funding from the State Health Fund. Thus, the costs determined by the FES-MG and the actual costs of the service provider were updated by the Extended Consumer Price Index (Índice de Preços ao Consumidor Amplo - IPCA)^25^ until December 2018 and compared.

The criterion for defining which cost would be considered was to observe whether the public funding for the service provider’s operations was sufficient. That is, even considering that the microcosting data could reflect, to some extent, some inefficiency, it mirrors the actual productivity of the service provider’s operations. That said, if the funds provided by the FES-MG to the center were sufficient to cover its costs, then the microcosting data would indicate greater efficiency than that estimated by the state government, and therefore, this cost would be considered.

The cost of DT was defined according to the mean expenditure of the SUS with service providers from SIGTAP (Table of Procedures, Medications, Orthoses, Prostheses, and Materials Management System)^26^, considering the main procedures related to hemodialysis and peritoneal dialysis.

To estimate the mean demand of patients, the mean number of consultations at the predialysis center was considered for the predialysis phase. For the DT phase, the demand predefined by the SUS through the High Cost/Complexity Procedure Authorization (APAC, for its acronym in Portuguese)^26^ was considered, that is, one monthly procedure per patient undergoing peritoneal dialysis and three sessions per week per patient undergoing hemodialysis.

### Cost analysis and sensitivity analysis

As parameters of demand variability, in the predialysis phase, the mean demand was considered per CKD progression stage according to KDIGO^21^. In the DT phase, 156 sessions per year were considered for hemodialysis, and 12 procedure per year were considered for peritoneal dialysis. 

In the probabilistic cost sensitivity analysis, the Monte Carlo simulation was used in a theoretical cohort of 10,000 patients (simulation with 10,000 interactions). According to the Ministry of Health’s Methodological Guidelines for Economic Evaluation in Health^27^^,^ 28, the Monte Carlo simulation is recommended to estimate cost variability, producing a probabilistic sensitivity measure from a stochastic perspective. Thus, the data have the power to provide potential information about likely cost variations.

Additionally, according to the guidelines^27^^,^ 28, the Gamma probability distribution was used to estimate the costs. For demand variability, there is no specific recommendation from the Ministry of Health, and therefore, the binomial distribution was used, establishing a 99% chance of the values approaching the mean for hemodialysis because the non-attendance of these patients at hemodialysis sessions severely compromises their health status. For peritoneal dialysis, 12 annual procedures were considered, with a 61.3% probability of the modality being automated peritoneal dialysis (APD) and 38.7% of it being continuous ambulatory peritoneal dialysis (CAPD), according to data on procedures approved by the Outpatient Information System of the SUS (SIA-SUS) from 2009 to 2018 29.

For probabilities of patient transition from predialysis to DT, it was established that 94.4% of patients would go on to hemodialysis and 5.6% would go on to peritoneal dialysis, according to data estimated from the procedures approved in the SIA-SUS in 201829. The probabilistic cost sensitivity analysis was performed using a stochastic decision tree model. For this purpose, PrecisionTree v7.5, Risk@ v7.5.1, and Microsoft Excel 2016 were used.

## Results

The Predialysis Outpatient Program served 37 cities of a microregion of Minas Gerais state. The distribution of patients relative to the population of each city was good at a certain level. The largest city had a population of 555,284 inhabitants, according to the Brazilian Institute of Geography and Statistics (Instituto Brasileiro de Geografia e Estatística - IBGE)^30^.

The mean participation of the cities’ populations as patients treated in the predialysis program was 0.75%. The highest participation rate of a city in the program was 2.03% and the lowest, 0.03%.

We conducted a retrospective cohort study of participants in the Predialysis Outpatient Program, which included the follow-up of 537 patients from 2011 to 2014. These patients had a mean age of 65 ± 13.3 years, and most were mixed-ethnicity females with a body mass index (BMI) of 29.9 ± 7.15, non-drinkers, ex-smokers or current smokers. Almost half (46.7%) had diabetes, and only 18.6% were using insulin. They were followed-up for a mean of 38.6 months (Table 1).

**Table 1:** Sociodemographic and clinical characteristics of the patients.

Variables	
Age (mean ± SD)	65.4 ± 13.3
Female sex (%)	51%
Ethnicity (%)	
White	23%
Mixed	47%
Black	30%
Education level (%)	
Illiterate	8%
Incomplete primary	67.5%
Complete primary	6.5%
Incomplete secondary	4.5%
Complete secondary	10%
Incomplete higher education	1%
Complete higher education	2.5%
Income (minimum wage) (dollar)	1.5 ± 1.8 (US$ 368.69 ± 442.43)
Alcohol consumption (%)	
Yes	18.4
Ex	24.4
Smoking status	
Present smoker	8%
Ex-smoker	45%
CKD stage at baseline (%)	
Stage 1	7.2%
Stage 2	19.3%
Stage 3	24.5%
Stage 3B	21%
Stage 4	19.7%
Stage 5	8%
Diabetes (%)	46,7%
BMI (mean ± SD)	29,9 ± 7,1
Drugs (%)	
ACEI	61,6%
ARB	69,2%
Beta-blockers	58,8%
Statins	76,1%
ASA	64,0%
Fibrates	16,0%
Biguanides	47,4%
Sulfonylureas	29,8%
Insulin	18,6%
Follow-up time (mean ± SD)	38,6 ± 9,5

* CKD - chronic kidney disease; BMI - body mass index; ACEI - angiotensin-converting enzyme inhibitors; BRAT- angiotensin receptor blockers; ASA-

Patients were seen by a specialist in the nephrology, cardiology, and endocrinology outpatient clinics, in addition to receiving multidisciplinary care. All patients had progressive CKD according to the KDIGO monitoring classification^21^ throughout the follow-up period (Figure 2). Reclassification of the CKD stage was performed every year.

**Figure 1:**
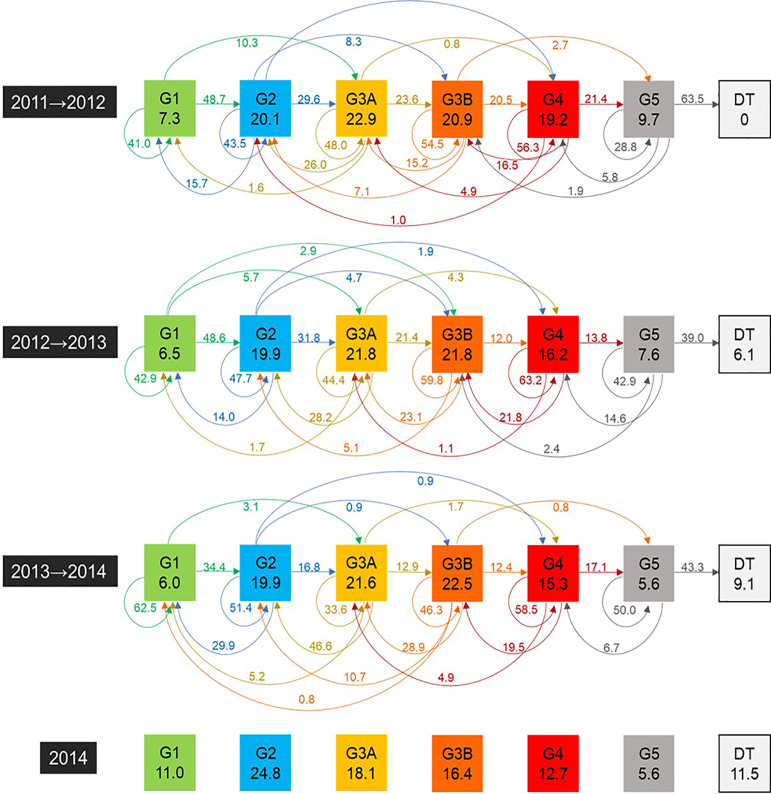
Odds of transition between stages of progressive chronic renal disease from 2011 and 2014 (in %) (1)

All patient exits from the predialysis phase were considered as entries into the DT phase. Therefore, with this survey, it was possible to define the odds of patients transitioning between progressive CKD stages (Figure 1).

**Figure 2:**
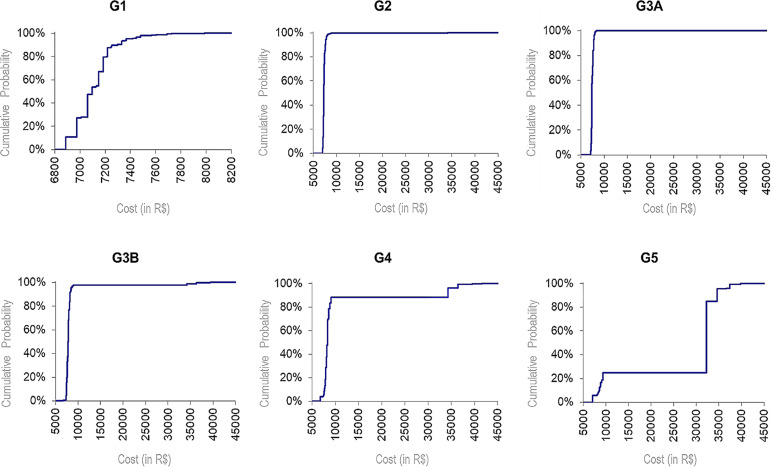
Cumulative probabilities for the progression of costs from predialysis to DT over a period of four years (in R$)

Figure 1 presents four annual transition timelines in which the first timeline, in each colored box, shows the CKD stage along with the percentage of patients identified in those risk strata at the beginning of the year. The horizontal arrows show the progression of the disease to the following stage. The curved arrows above the boxes show the most severe jumps in disease progression, and the curved arrows below the boxes show backward jumps in disease progression. Some of the jumps occurred at the thresholds between stages.

The results of the analysis reveal interesting information, as summarized in Figure 2. The average cost of a population with CKD tends to increase substantially as the disease progresses. Stage G1 recorded an average cost of R$ 7,110.78 (US$1,832.06), and stage G5 reached an average cost of R$ 26,814.08 (US$6,908.53), accumulated over the four years. The average cost of this last stage increases because the patient has greater odds of being referred to DT within a period of four years. Details on the collection of data regarding the cost of predialysis care can be found in the supplementary material.

According to Table 2, the standard deviation increases starting in stage G3B. The variation in the standard deviation of CKD stage G2 was due to the higher demand from patients with diabetes than that demanded from patients in stage G1 and from patients with hypertension than from those in stage G3A, which in turn had the lowest mean demand from patients with hypertension. Thus, the odds of stage G3A incurring costs with DT was slightly increased compared to stages G1 and G2.

**Table 2:** Results of the probabilistic cost sensitivity analysis.

	G1	G2	G3A	G3B	G4	G5	Overall
Mean	R$ 7.110,78 (US$ 1.832,06)	R$ 7,440,73 (US$ 1.917,07)	R$ 7.449,12 (US$ 1.919,23)	R$ 8.422,92 (US$ 2.170,13	R$ 11.328,92 (US$ 2.918,85)	R$ 2.6814,08 (US$ 6.908,53)	R$ 10.245,32 (US$ 2.639,66)
Standard deviation	R$ 155,60 (US$ 40,09)	R$ 1.903,83 (US$ 490,51)	R$ 675,63 (US$ 174,07)	R$ 4.166,39 (US$ 1.073,45)	R$ 8.723,15 (US$ 2.247,48)	R$ 10.663,21 (US$ 2.747,33	R$ 7.851,68 (US$ 2.022,95)
Minimum	R$ 6.885,92 (US$ 1.774,13)	R$ 6.492,01 (US$ 1.672,64)	R$ 7.007,85 (US$ 1.805,54)	R$ 6.640,69 (US$ 1.710,94)	R$ 6.762,86 (US$ 1.742,42	R$ 7.024,55 (US$ 1.809,84)	R$ 6.492,01 (US$ 1.672,64)
Maximum	R$ 7.986,23 (US$ 2.057,62)	R$ 41.326,48 (US$ 10.647,59)	R$ 41.360,91 (US$ 10.656,46)	R$ 41.475,15 (US$ 10.685,89)	R$ 41.597,32 (US$ 10.717,37	R$ 41.859,02 (US$ 10.784,79)	R$ 41.859,02 (US$ 10.784,79)
Mode	R$ 7.060,92 (US$ 1.819,22)	R$ 7.270,36 (US$ 1.873,18)	R$ 7.339,21 (US$ 1.890,92)	R$ 7.830,62 (US$ 2.017,53)	R$ 8.319,29 (US$ 2.143,43)	R$ 32.248,32 (US$ 8.308,64)	R$ 32.248,32 (US$ 8.308,64)
Risk of predialysis costs	100,00%	99,53%	99,95%	97,73%	88,30%	24,94%	89,91%
Minimum predialysis cost	R$ 6.885,92 (US$ 1.774,13)	R$ 6.492,01 (US$ 1.672,64)	R$ 7.007,85 (US$ 1.805,54)	R$ 6.640,69 (US$ 1.710,94)	R$ 6.762,86 (US$ 1.742,42)	R$ 7.024,55 (US$ 1.809,84)	R$ 6.492,01 (US$ 1.672,64)
Maximum predialysis cost	R$ 7.986,23 (US$ 2.057,62)	R$ 8.833,53 (US$ 2.275,92)	R$ 8.606,27 (US$ 2.217,37)	R$ 8.982,20 (US$ 2.314,22)	R$ 9.104,37 (US$ 2.345,7)	R$ 9.366,07 (US$ 2.413,13)	R$ 9.366,07 (US$ 2.413,13)
Risk of DT costs	0,00%	0,47%	0,05%	2,27%	11,70%	75,06%	10,09%
Minimum DT cost		R$ 34.057,30 (US$ 8.774,71)	R$ 36.171,55 (US$ 9.319,44)	R$ 34.205,97 (US$ 8.813,02)	R$ 34.328,14 (US$ 8.844,5)	R$ 32.248,32 (US$ 8.308,64)	R$ 32.248,32 (US$ 8.308,64)
Maximum DT cost		R$ 41.326,48 (US$ 10.647,59)	R$ 41.360,91 (US$ 10.656,46)	R$ 41.475,15 (US$ 10.685,89)	R$ 41.597,32 (US$ 10.717,37)	R$ 41.859,02 (US$ 10.784,79)	R$ 41.859,02 (US$ 10.784,79)

In general, the average costs were impacted by stages G3B to G5, causing a greater dispersion in the costs, denoting a probable risk of higher costs (Table 2). In fact, this event may occur over a period of four years. There was a 10.09% chance of a patient migrating to DT incurring a cost between R$ 32,248.32 (US$ 8,308.64) and R$ 41,859.00 (US$ 10,784.79); however, there was a 89.91% chance of patient costs ranging from R$ 6,492.01 (US$ 1,672.64) to R$ 9,366.07 (US$ 2,413.13). Notably, the risk of incurring costs with DT in stage G3A over a period of four years was practically nil. Furthermore, the risk for stage G3B was also very low.

A predialysis program can generate an average cost reduction of R$ 33,023.12 ± 1,676.80 (US$ 8,508.26 ± 432.02) for each year of DT avoided, which covers the program’s operational cost, thus minimizing cost. These results signal to public health policy makers the real possibility of achieving visible results for the care of CKD in the medium term (4 years) for a program that disbursed R$ 24 billion (US$ 6.8 billion) for DT in Brazil between 2009 and 2018. 

## Discussion

We demonstrated that in a multidisciplinary care model from the perspective of the service provider in the reality of the Brazilian public health system there is an increase in cost as the severity of CKD progresses. In addition, the cost of DT is very high compared to predialysis costs, even in more advanced disease stages. By showing that each year of DT avoided generates a reduction in the monthly cost per patient, we emphasize that this is a cost-minimizing strategy.

Kidney diseases and some of the main related diseases accounted for 12.97% of the expenditures of the SUS in Brazil in the 2013-2015 triennium, and renal replacement therapy (RRT) accounted for more than 5% of the SUS expenditures on medium- and high-complexity healthcare^22^. It would be plausible for public health actions to focus on avoiding late disease diagnosis, thus allowing easier access to specialized multidisciplinary care^10^^,^ 11, mitigating the impairment of individuals’ productive capacity 12 and the high costs of DT^13^. 

Specialized care to patients with CKD during disease progression is still an underexplored field. A study conducted in Taiwan reported that patients with CKD who received high-quality nephrological care during the predialysis phase incurred lower costs during the dialysis phase and had higher survival rates. These data is useful for health managers and physicians and provide evidence that financial incentives can help improve the quality of services provided in the predialysis phase. These findings are in agreement with our study, which showed that adequate multidisciplinary predialysis care, delaying the progression of CKD to ESRD, is a cost-minimizing strategy^23^.

There is implicit, rather than estimated, reduction in the social cost of CKD when investing in prevention14. The results presented here echo evidence that in Brazil, the SUS strategies for combating CKD are more focused on treatment than prevention, which agrees with studies that indicate that preventive actions improve quality of life and seek greater economic balance between costs and quality in healthcare services^15^^,^ 16. 

A retrospective study conducted in the Lombardy Region, Italy, evaluated the cost in the first year after starting DT and in the two years prior to it. The costs of drugs, hospitalizations, and diagnostic and outpatient procedures covered by the public health system were estimated. The results highlight a significant economic burden related to CKD and an increase in the direct health costs associated with the start of dialysis, indicating the importance of prevention and early diagnosis programs^24^. Although our study had a different approach, we observed a similar finding, with lower cost in predialysis care.

In Brazil, a study estimated the cost incurred by the SUS over a period of seven years and concluded that the cost of predialysis and dialysis care attributed to diabetes was high^31^. However, in that study, the cost was evaluated from the perspective of the payer, the SUS, and did not have access to all the variables necessary for a realistic result^31^. Our study used the perspective of the service provider and took into account most of the variables associated with predialysis care costs using the methodology suggested by the Ministry of Health for this approach^27^^,^ 28.

As observed in studies conducted in various parts of the world and in our study conducted in Brazil, which is facing legislative changes toward fiscal austerity and an increasingly restrictive public health funding^20^, public health managers should consider predialysis care as an economic option for public health actions and services to combat CKD.

We believe that the main limitation of our study was not having determined the cost of complications associated with the need for hospitalization, because these are funded by the SUS. Another limitation is that fatal events that may occur more frequently in individuals with more advanced CKD were not taken into account, however this data do not interfere with cost analysis during predialysis care.

DT will continue to be the therapeutic option for patients with ESRD^21^, but certainly, shrewd management in the combating of CKD will need a greater focus of the public budget and public policies that are conducive to and that support the provision of predialysis care services.

We conclude that the earlier the adherence of patients with CKD to predialysis programs, the higher is the cost-minimizing effects on DT, complying with a short- and medium-term strategy, screening actions, and more effective awareness campaigns .

Preventive and planned care for combating CKD in Brazil and in the world must be based on important information for health actions and services to guarantee the fundamental right to life so that the future is not a trade-off between savings and health provision.

## Supplementary Material

The following online material is available for this article:

Supplementary material
